# Associations of depression with hypertension and citizenship among U.S. adults: A cross-sectional study of the interactions of hypertension and citizenship

**DOI:** 10.1016/j.pmedr.2023.102523

**Published:** 2023-11-23

**Authors:** Emmanuel A. Odame, Paul H. Atandoh, Lohuwa Mamudu, David Adzrago, Ishmael Tagoe, Saanie Sulley, Maureen Boms, Erasmus Tetteh-Bator, Timothy S. McNeel, Faustine Williams

**Affiliations:** a Department of Environmental Health Sciences, School of Public Health, University of Alabama at Birmingham, Birmingham, AL, USA; b Department of Mathematics, Mercer University, Macon, GA, USA; c Department of Public Health, California State University, Fullerton, CA, USA; d Division of Intramural Research, National Institute on Minority Health and Health Disparities, National Institutes of Health, Bethesda, MD, USA; e Division of Health Services, College of Nursing and Advanced Health Professions, The Chicago School of Professional Psychology, IL, USA; f National Healthy Start Association, Washington, DC, USA; g Department of Epidemiology, School of Public Health, University of Alabama at Birmingham, Birmingham, AL, USA; h Department of Mathematics and Statistics, College of Arts and Sciences, University of South Florida, Tampa, FL, USA; i Information Management Services, Inc, Calverton, MD, USA

**Keywords:** Hypertension, Depression, Comorbidity, Citizenship, Immigrant

## Abstract

With the increasing prevalence of hypertension-related cardiovascular deaths and depression, this study examined the associations of depression with hypertension, citizenship status, and interaction of hypertension and citizenship status among U.S. adults. Data from the 2015–2018 National Health Interview Survey (NHIS), including 63,985 individuals, were analyzed. Depression status was the outcome, with hypertension and U.S. citizenship status as the main independent variables. Using odds ratio (OR) estimates, we evaluated the associations between hypertension and depression, and citizenship status. The result indicates that a higher proportion of U.S. adults with hypertension reported depression compared to those who did not have hypertension (42.9 % vs. 37.5 %). In terms of U.S. citizenship status, a higher proportion of U.S. citizens reported depression than non-citizens (39.6 % vs. 31.6 %). However, non-citizens with hypertension were more likely to report depression compared to U.S. citizens without hypertension (OR = 1.46; 95 % CI = 1.15, 1.86). While hypertension marginally increased the odds of depression among the general U.S. population, being a non-U.S. citizen with hypertension significantly increased the risk of depression by 46 %. The findings imply that the healthy immigrant paradox, in the context of hypertension-depression prevention and control, may not apply to non-citizens with hypertension. We therefore recommend community-based screenings and more tailored interventions to address these health disparities while taking into consideration the unique cultural norms, behaviors and healthcare barriers encountered by specific immigrant communities.

## Highlights

• Depression is common among patients with hypertension.
• Disparities exist in hypertension and depression by citizenship status in the U.S.
• U.S. citizens reported a higher prevalence of depression than non-citizens.
• Non-U.S. citizens with hypertension had higher odds of depression compared to U.S. citizens without hypertension.

## 1 Introduction

Hypertension is prevalent in the United States (U.S.) with an estimated 47 % of adults having this condition (Centers for Disease Control and Prevention, 2021a, b). It increases the risk for heart disease and stroke, the two leading causes of death in the United States (Kochanek et al., 2019, Murphy et al., 2021). Hypertension-related cardiovascular death has significantly increased over time (Nambiar et al., 2020). In 2019, more than half a million deaths in the U.S. had hypertension as a primary or contributing cause of death (Centers for Disease Control and Prevention, 2021b).

Depression is a common comorbidity among patients with hypertension (Marazziti et al., 2014). The prevalence of depression is higher in hypertensive patients than in the general population (Kessing et al., 2020). A systematic review and *meta*-analysis using 41 studies found that approximately one-third of patients with hypertension also present with depression (Li et al., 2015). Existing evidence indicates that both hypertension and depression share common biological pathways including activation of the renin-angiotensin-aldosterone system (DeJean et al., 2013, Hafner et al., 2013, Rubio-Guerra et al., 2013). While previous studies have found the use of antidepressants to increase blood pressure through multiple effects on various pathways and systems in the body (Breeden et al., 2018, Calvi et al., 2021), antihypertensive medications can also affect the risk of depression (Kessing et al., 2020, Li et al., 2021). A recent nationwide study was conducted using the Danish population-based register to investigate whether commonly used antihypertensive medications were associated with depression (Kessing et al., 2020). Contrary to previous findings, the authors reported that no antihypertensive drug was associated with an increased risk of depression but rather the continued use of angiotensin agents, calcium antagonists, and β-blockers significantly reduce the rates of depression (Kessing et al., 2020).

In general, depression varied by sociodemographic characteristics (e.g., age, sex), with individuals with lower socioeconomic status (e.g., females, younger adults, unemployed) having higher burdens of depression (Gabriel et al., 2021, Mann et al., 2022, Villarroel and Terlizzi, 2020). Several other studies have examined the association between hypertension and depression and have reported mixed findings (Ginty et al., 2013, Grimsrud et al., 2009, Ho et al., 2015, Maatouk et al., 2016, Rubio-Guerra et al., 2013, Wang et al., 2021, Wiehe et al., 2006). These studies, however, did not delineate differences found in the current literature among underserved populations including minorities and immigrants (Commodore-Mensah et al., 2021, Gabriel et al., 2021, Guadamuz et al., 2020). For instance, citizenship status has been found to contribute to disparities in hypertension and other cardiovascular conditions (Guadamuz et al., 2020). A study on the association between immigration status and anxiety, depression, and use of anxiolytic and antidepressant medications in the Hispanic Community also reported that undocumented Hispanic non-citizens in the U.S. were less likely to use medication for depression compared to citizens and documented non-citizens (Ross et al., 2019).

The U.S. immigrant population has been increasing rapidly and is expected to be a major driver of population growth (Guadamuz et al., 2020, Jordan and Gebeloff, 2022). New immigrants are known to have lower incidence of chronic health conditions, a phenomenon described as the healthy immigrant paradox (Bustamante et al., 2021, Stanek et al., 2020). Nonetheless, disparities in healthcare resources, social disadvantages, neighborhood conditions, and low socioeconomic status associated with excess burden of cardiovascular diseases have also been well-documented among immigrants (Chang, 2019, de Mestral and Stringhini, 2017, Hamad et al., 2020). This poses a major health risk of further increase in the prevalence of hypertension and other chronic conditions in this population. For example, immigrants are more likely to experience limited access to care including inequitable access/treatment and low adherence to medications (Cho et al., 2020, Guadamuz et al., 2021, Hacker et al., 2015). Yet, no study to date has investigated citizenship and hypertension status disparities in depression among U.S. adults using nationally representative data.

This study aims to: (1) estimate the prevalence of hypertension and depression among U.S. adults, (2) determine the associations of depression with hypertension, citizenship status, and the interaction between hypertension and citizenship status, and (3) sociodemographic and other factors among U.S. adults.

## 2 Methods

### 2.1 Study participants and design

Data from the 2015–2018 National Health Interview Survey (NHIS) (Blewett et al., 2021) were analyzed. The NHIS is a cross-sectional household interview survey that targets the civilian noninstitutionalized population of the U.S. Thus, weighted estimates cover only the civilian noninstitutionalized household population. Individuals in long-term care institutions, correctional facilities, and U.S. nationals living in foreign countries were excluded. Active-duty Armed Forces personnel are also excluded from the survey, unless at least one other family member is a civilian eligible for the survey but they were not weighted (National Center for Health Statistics, 2019c).

Analyses were restricted to adults (ages 18 and older) who received questions about depression. The analyses were weighted using the NHIS sampling weights. The sampling weights are derived from person-level weights indicating the number of population units that a sampled unit represents. The person-level weights are adjusted for nonresponse and ratio-adjusted to create final sampling weights, which are further adjusted according to a quarterly post stratification by age/sex/race/ethnicity classes based on population estimates produced by the U.S. Census Bureau (Parsons et al., 2014). Also, oversampling was applied to minority household populations. There were 72,159 participants in the 2015–2018 datasets. However, we performed a complete case analysis on 63,985 individuals. The remaining 8,174 participants were excluded due to incomplete or missing data. In this study, depression was the outcome of focus, with hypertension and citizen status as primary independent variables or exposures of interest, and sociodemographic factors as secondary independent variables.

### 2.2 Measures

#### 2.2.1 Outcome

To assess the outcome, depression, of this study, respondents were categorized as depressed if they reported feeling depressed “daily, weekly, monthly or a few times per year or more” or if they reported “yes” that they take medication for depression. Otherwise, respondents were categorized as not depressed. To address the issue of under identification of depression, especially among non-citizens, we included those who use medication for depression. For instance, it has been reported that immigrants and other racial/ethnic minorities may not report being depressed even if they have agreed to treatment due to the stigma attached to this condition (Chen et al., 2016). Hence, it was essential to include individuals taking medication for depression to minimize sampling error.

#### 2.2.2 Primary independent variables

Independent variables in this study include hypertension and citizenship status. Hypertension was categorized into two: having hypertension or not having hypertension as the reference [Ref] category. Respondents were categorized as having hypertension if they reported that “they had hypertension or high blood pressure in the past 12 months” or were “taking any medication prescribed by a doctor for high blood pressure at the time of the interview.” Individuals’ citizenship status was classified as being a U.S. citizen [Ref] or non-U.S. citizen. Respondents were asked whether they are U.S. citizens or non-citizens, and they responded yes or no.

#### 2.2.3 Secondary independent variables

The secondary independent variables include self-reported age (18–29, 30–44, 45–64, and ≥ 65 [Ref]). Sex was categorized as (female [Ref] and male); sexual orientation (heterosexual [Ref] and non-heterosexual [gay, bisexual, something else]); race/ethnicity (non-Hispanic White [Ref], non-Hispanic Black, Hispanic and non-Hispanic); marital status (never married [Ref], married/living with partner, divorced/separated/widowed); education (bachelor's degree or more [Ref], less than high school graduate, high school graduate/GED and Technical/some college); employment status (employed [Ref] and not employed); annual household income (≥ $65,000 [Ref], $35,000 - $64,999 and <$35,000); alcohol drinking status (lifetime abstainer i.e. < 12 drinks in life [Ref], former drinker, i.e. no drinks past year and current drinker i.e. 1 + drinks past year); BMI category (<25.00, i.e. underweight/normal [Ref], 25.00–29.99, i.e. overweight and ≥ 30.00, i.e. obese); smoking status (never [Ref], former smoker and current smoker]; leisure-time physical activity (physically active, i.e. having at least 150 min of moderate activity or 75 min of vigorous activity per week or an equivalent combination [Ref] and Inactive/Insufficient physical activity); number of known chronic conditions (none, i.e. 0 [Ref], 1–2 chronic conditions and ≥ 3 chronic conditions); general health status (very good /excellent [Ref], good, poor/fair); health insurance (has coverage [Ref] and does not have coverage); years lived in the U.S. (<5 years [Ref], 5 to < 15 years and ≥ 15 years).

The number of chronic conditions counted how many of the following conditions the respondent ever had: asthma, arthritis (including arthritis, rheumatoid arthritis, gout, lupus, or fibromyalgia), cancer, emphysema, coronary heart disease, diabetes, hepatitis, hypertension, stroke; plus, the number of the following conditions they had in the past 12 months: weak or failing kidneys, chronic bronchitis. Missing values for income were imputed and analyzed using a multiple-imputation methodology (National Center for Health Statistics, 2019b, National Center for Health Statistics, 2019).

### 2.3 Statistical analyses

We first assessed the prevalence of depression and hypertension along with descriptive characteristics of sociodemographic and other factors using frequencies and percentages. Then two multivariable logistic regression models were used to examine (1) the associations of depression with hypertension status, citizenship status, and sociodemographic factors (2) interaction of hypertension status and citizenship status, adjusting for the sociodemographic factors. Additionally, predicted marginal proportions for depression by citizenship status and hypertension were also calculated. Analyses were weighted, and Taylor series linearization methods were used to account for the stratified, multistage, cluster sample design of the NHIS (National Center for Health Statistics, 2019a, National Center for Health Statistics, 2019). Results are reported based on odds ratio (OR) along with 95 % confidence interval (CI) and p-values at 0.05 statistical level of significance. Analyses were conducted using SUDAAN 11.0.3.

## 3 Results

### 3.1 Descriptive statistics

Descriptive statistics of depression, citizenship status, hypertension, and other factors are presented in Table 1. A little over 92 % of the respondents identified as U.S. citizens, while 7.9 % were non-citizens. A higher proportion of U.S. adults with hypertension reported as feeling depressed or taking medications for depression compared to those who did not have hypertension (42.9 % vs. 37.5 %). In terms of U.S. citizenship status and depression, 39.6 % of citizens had depression while 31.6 % of non-citizens reported being depressed. The highest prevalence of depressed individuals were aged 45–64 (40.7 %), females (43.8 %), non-heterosexuals (61.8 %), non-Hispanic Whites (41.8 %), divorced/separated or widowed (45 %), had less than high school education (40.3 %), not employed (44.1 %), had annual household income < $35,000 (46.2 %), resided in the West (42.1 %), former alcohol drinkers (43.3 %), obese (43.0 %), current smokers (49.9 %), inactive or had insufficient physical activity (40.7 %), had 3 or more chronic conditions (56.6 %), had poor or fair health (61.4 %), had no health insurance coverage (41.8 %) and had lived in the U.S. for at least 15 years (39.5 %).

**Table 1:** Weighted descriptive statistics of depression (outcome) and U.S. citizenship status, hypertension, and other factors among U.S. adults, NHIS 2015–2018.

	**Total Sample**	**Depressed**	**Not Depressed**
	n (%)	n (%)	n (%)
	63,985 (1 0 0)	26,290 (39)	37,695 (61)
**U.S. Citizenship**			
Citizens	59,895 (92.1)	24,953 (39.6)	34,942 (60.4)
Non-Citizens	4090 (7.9)	1337 (31.6)	2753 (68.4)
**Hypertension (had hypertension in past 12 months or taking medicine for hypertension)**			
Yes	19,941 (26.9)	8834 (42.9)	11,107 (57.1)
No	44,044 (73.1)	17,456 (37.5)	26,588 (62.5)
**Sociodemographic and Other Factors**			
**Age**			
18–29	10,328 (21.1)	4265 (39.3)	6063 (60.7)
30–44	15,025 (25.6)	6243 (38.7)	8782 (61.3)
45–64	21,279 (33.5)	9246 (40.7)	12,033 (59.3)
≥65	17,353 (19.9)	6536 (36.1)	10,817 (63.9)
**Sex**			
Male	29,478 (49.1)	10,518 (34.0)	18,960 (66.0)
Female	34,507 (50.9)	15,772 (43.8)	18,735 (56.2)
**Sexual Orientation**			
Heterosexual	61,805 (96.7)	24,933 (38.2)	36,872 (61.8)
Non-Heterosexual	2180 (3.3)	1357 (61.8)	823 (38.2)
**Race/Ethnicity**			
Non-Hispanic White	43,604 (64.6)	18,946 (41.8)	24,658 (58.2)
Non-Hispanic Black	7086 (11.4)	2525 (32.6)	4561 (67.4)
Hispanic	8234 (15.7)	2916 (33.5)	5318 (66.5)
Non-Hispanic Other	5061 (8.3)	1903 (36.4)	3158 (63.6)
**Marital Status**			
Never Married/Single	14,233 (22.3)	6443 (42.8)	7790 (57.2)
Married/Living with Partner	32,654 (60.9)	12,013 (35.9)	20,641 (64.1)
Divorced/Separated/Widowed	17,098 (16.9)	7834 (45.0)	9264 (55.0)
**Educational Level**			
Less than High School Graduate	7412 (11.7)	3084 (40.3)	4328 (59.7)
High School Graduate/GED	15,638 (24.5)	6371 (39.2)	9267 (60.8)
Technical/Some College	19,968 (30.8)	8537 (40.2)	11,431 (59.8)
Bachelor’s Degree or More	20,967 (33.1)	8298 (37.2)	12,669 (62.8)
**Employment Status**			
Employed	36,689 (61.7)	14,077 (35.8)	22,612 (64.2)
Not Employed	27,296 (38.3)	12,213 (44.1)	15,083 (55.9)
**Annual Household Income (with imputed values)**			
<$35,000	22,347 (27.7)	10,642 (46.2)	11,704 (53.8)
$35,000–64,999	15,772 (24.0)	6372 (39.2)	9400 (60.8)
≥$65,000	25,867 (48.3)	9276 (34.8)	16,591 (65.2)
**Alcohol Drinking Status**			
Lifetime Abstainer (<12 drinks in life)	11,683 (19.4)	3922 (32.4)	7761 (67.6)
Former Drinker (no drinks past year)	9801 (13.5)	4365 (43.3)	5436 (56.7)
Current Drinker (1 + drinks past year)	42,501 (67.2)	18,003 (40.0)	24,498 (60.0)
**BMI**^1^**Category**			
<25.00, Underweight/Normal	21,978 (34.6)	8824 (37.9)	13,154 (62.1)
25.00–29.99, Overweight	22,052 (34.4)	8498 (36.4)	13,554 (63.6)
≥30.00, Obese	19,955 (31.0)	8968 (43.0)	10,987 (57.0)
**Smoking Status**			
Never	38,340 (63.1)	14,359 (35.6)	23,981 (64.4)
Former	15,730 (22.3)	6745 (41.5)	8985 (58.5)
Current	9915 (14.5)	5186 (49.9)	4729 (50.1)
**Leisure-Time Physical Activity**^2^			
Inactive /Insufficient	30,975 (47.3)	13,388 (40.7)	17,587 (59.3)
Physically Active	33,010 (52.7)	12,902 (37.4)	20,108 (62.6)
**Number of known Chronic Conditions**			
0	32,254 (54.9)	11,207 (32.9)	21,047 (67.1)
1–2	26,141 (38.1)	11,895 (44.5)	14,246 (55.5)
≥3	5590 (7.0)	3188 (56.6)	2402 (43.4)
**General Health Status**			
Poor/Fair	8985 (12.5)	5542 (61.4)	3443 (38.6)
Good	17,303 (26.2)	7825 (43.3)	9478 (56.7)
Very Good/Excellent	37,697 (61.3)	12,923 (32.6)	24,774 (67.4)
**Health insurance**			
Has Coverage	58,141 (90.0)	23,800 (38.8)	34,341 (61.2)
Does not Have Coverage	5844 (10.0)	2490 (41.0)	3354 (59.0)
**Years Lived in the U.S.**			
<5	1040 (1.9)	346 (29.7)	694 (70.3)
5 to < 15	1902 (3.8)	576 (30.0)	1326 (70.0)
≥15	61,043 (94.2)	25,368 (39.5)	35,675 (60.5)

NHIS = National Health Interview Surveys.

1 BMI = Body Mass Index.

2 Leisure-time physical activity (goal:150 min/week moderate activity or 75 min vigorous activity, or equivalent combination).

### 3.2 Multivariable logistic regression

Table 2 presents the results of the multivariable logistic regression, which depicts the associations between depression, U.S. citizenship status, hypertension, intersection of citizenship and hypertension status, and other factors. Both models 1 (without interaction) and 2 (with interaction) indicate that individuals with hypertension had slightly higher odds of depression (Model 1: OR = 1.05; 95 % CI = 0.99, 1.11; and Model 2: OR = 1.02; 95 % CI = 0.97, 1.08). Nevertheless, this association was not statistically significant.

**Table 2:** Multivariable logistic regression depicting the association between depression (outcome) and U.S. citizenship status, hypertension, and other factors among U.S. adults, NHIS 2015–2018.

	**Model 1** **(without interaction)**		**Model 2** **(with interaction)**	
	OR	95 % CI	OR	95 % CI
**U.S. Citizenship**				
Citizens	Ref	–	Ref	–
Non-Citizens	1.01	(0.89, 1.14)	0.92	(0.80, 1.07)
**Hypertension (had hypertension in past 12 months or taking medicine for hypertension)**				
No	Ref	–	Ref	–
Yes	1.05	(0.99, 1.11)	1.02	(0.97, 1.08)
Interaction Term (U.S. citizenship* hypertension)				
U.S. Citizen, No	–	–	Ref	–
U.S. Citizen, Yes	–	–	1.02	(0.97, 1.08)
Non-U.S Citizen, No	–	–	0.92	(0.80, 1.07)
Non-U.S. Citizen, Yes	–	–	1.46	(1.15, 1.86)
**Sociodemographic and Other Factors**				
**Age**				
≥65	Ref	–	Ref	–
18–29	2.20	(2.00, 2.42)	2.20	(1.99, 2.42)
30–44	2.16	(1.99, 2.36)	2.16	(1.99, 2.36)
45–64	1.80	(1.67, 1.93)	1.79	(1.67, 1.92)
**Sex**				
Female	Ref	–	Ref	–
Male	0.63	(0.60, 0.66)	0.63	(0.60, 0.66)
**Sexual Orientation**				
Heterosexual	Ref	–	Ref	–
Non-Heterosexual	2.21	(1.94, 2.52)	2.21	(1.94, 2.52)
**Race/Ethnicity**				
Non-Hispanic White	Ref	–	Ref	–
Non-Hispanic Black	0.54	(0.49, 0.58)	0.54	(0.49, 0.58)
Hispanic	0.68	(0.63, 0.75)	0.68	(0.63, 0.74)
Non-Hispanic Other	0.85	(0.77, 0.93)	0.85	(0.77, 0.93)
**Marital Status**				
Never Married/Single	Ref	–	Ref	–
Married/Living with Partner	0.73	(0.69, 0.79)	0.73	(0.69, 0.79)
Divorced/Separated/Widowed	0.90	(0.83, 0.96)	0.90	(0.83, 0.97)
**Educational Level**				
Bachelor’s Degree or More	Ref	–	Ref	–
Less than High School Graduate	0.82	(0.75, 0.90)	0.82	(0.75, 0.90)
High School Graduate/GED	0.85	(0.79, 0.91)	0.85	(0.79, 0.91)
Technical/Some College	0.90	(0.85, 0.95)	0.89	(0.85, 0.95)
**Employment Status**				
Employed	Ref	–	Ref	–
Not Employed	1.29	(1.22, 1.37)	1.29	(1.22, 1.37)
**Annual Household Income (with imputed values)**				
≥$65,000	Ref	–	Ref	–
<$35,000	1.29	(1.20, 1.39)	1.29	(1.20, 1.39)
$35,000–64,999	1.11	(1.04, 1.18)	1.11	(1.04, 1.18)
**Alcohol Drinking Status**				
Lifetime Abstainer (<12 drinks in life)	Ref	–	Ref	–
Former Drinker (no drinks past year)	1.38	(1.26, 1.51)	1.38	(1.26, 1.51)
Current Drinker (1 + drinks past year)	1.53	(1.42, 1.64)	1.53	(1.42, 1.64)
**BMI**^2^**Category**				
<25.00, Underweight/Normal	Ref	–	Ref	–
25.00–29.99, Overweight	1.01	(0.96, 1.07)	1.01	(0.96, 1.07)
≥30.00, Obese	1.08	(1.02, 1.15)	1.08	(1.02, 1.15)
**Smoking Status**				
Never	Ref	–	Ref	–
Former	1.13	(1.07, 1.21)	1.14	(1.07, 1.21)
Current	1.36	(1.27, 1.46)	1.36	(1.27, 1.46)
**Leisure-Time Physical Activity**				
Physically Active	Ref	–	Ref	–
Inactive /Insufficient	0.94	(0.89, 0.99)	0.94	(0.89, 0.99)
**Number of known Chronic Conditions**				
0	Ref	–	Ref	–
1–2	1.48	(1.40, 1.56)	1.48	(1.40, 1.56)
≥3	1.91	(1.73, 2.11)	1.92	(1.73, 2.12)
**General Health Status**				
Very Good/Excellent	Ref	–	Ref	–
Poor/Fair	2.86	(2.64, 3.09)	2.85	(2.63, 3.08)
Good	1.51	(1.43, 1.60)	1.51	(1.43, 1.60)
**Health insurance**				
Has Coverage	Ref	–	Ref	–
Does not Have Coverage	1.10	(1.01, 1.20)	1.11	(1.02, 1.21)
**Years Lived in the U.S.**				
<5	Ref	–	Ref	–
5 to < 15	1.06	(0.85, 1.33)	1.03	(0.83, 1.29)
≥15	1.23	(0.99, 1.52)	1.17	(0.94, 1.46)

OR = Odds ratios for model.

NHIS = National Health Interview Surveys.

Ref = Reference.

2 BMI = Body Mass Index.

For model 1 (without the interaction term), the highest odds of depression were reported among young adults aged 18–29 years compared to adults age ≥ 65 years (OR = 2.20; 95 % CI = 2.00, 2.42), non-heterosexuals compared to heterosexuals (OR = 2.21; 95 % CI = 1.94, 2.52), unemployed individuals compared to those employed (OR = 1.29; 95 % CI = 1.22, 1.37), individuals having income less than $35,000 compared to those with income ≥ $65,000 (OR = 1.29; 95 % CI = 1.20, 1.39), current drinkers compared to lifetime abstainers (OR = 1.53; 95 % CI = 1.42, 1.64), obese adults compared to normal/underweight adults (OR = 1.08; 95 % CI = 1.02, 1.15), current smokers compared to non-smokers (OR = 1.36; 95 % CI = 1.27, 1.46), individuals having 3 + chronic conditions compared to those with none (OR = 1.91; 95 % CI = 1.73, 2.11), individuals with poor/fair health compared to those with very good/excellent health (OR = 2.86; 95 % CI = 2.64, 3.09), and individuals without health insurance compared to those with health insurance (OR = 1.10; 95 % CI = 1.01, 1.20). The odds of depression also increased with length of stay in the U.S. (OR = 1.06 [95 % CI = 0.85, 1.33] and 1.23 [95 % CI = 0.99, 1.52] for adults living in the U.S. for 5 to < 15 years and ≥ 15 years, respectively, compared to < 5 years), but were not statistically significant. On the other hand, lower odds of depression were associated with individuals who were males (OR = 0.63; 95 % CI = 0.60, 0.66), non-Hispanic Blacks (OR = 0.54; 95 % CI = 0.49, 0.58), Hispanics (OR = 0.68; 95 % CI = 0.63, 0.75), non-Hispanics other (OR = 0.85; 95 % CI = 0.77, 0.93), those married/living with a partner (OR = 0.73; 95 % CI = 0.69, 0.79), and divorced/separated/widowed (OR = 0.90; 95 % CI = 0.83, 0.96). The odds of depression also decreased with lower levels of education (OR = 0.82 [95 % CI = 0.75, 0.90] for less than high school graduates, OR = 0.85 [95 % CI = 0.79, 0.91] for high school graduates/GED and OR = 0.90 [95 % CI = 0.85, 0.95] for technical/some college degree holders compared to those with bachelor’s degree or more) and being physically inactive/insufficiently active (OR = 0.94; 95 % CI = 0.89, 0.99).

For model 2 (with interaction model), there was a statistically significant interaction between hypertension and citizenship status (Wald F = 3.98, *p* = 0.009). Higher odds of depression was observed among non-US citizens with hypertension relative to U.S. citizens without hypertension (OR = 1.46; 95 % CI = 1.15, 1.86). Non-U.S. citizens without hypertension had lower odds of depression while U.S. citizens with hypertension had higher odds of depression compared to U.S. citizens without hypertension, although not statistically significant.

### 3.3 Predicted marginal results

**Fig. 1:**
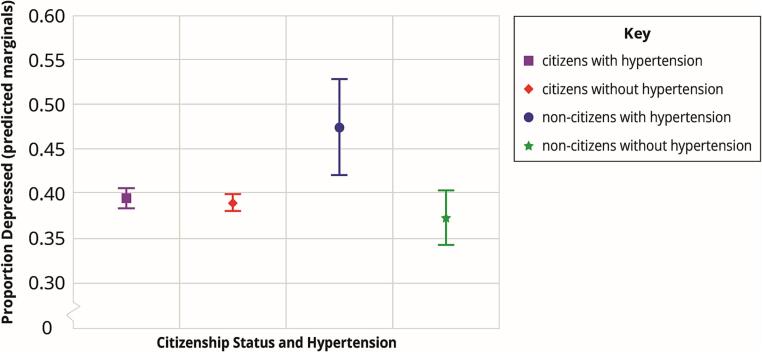
U.S. Adults with Depression by Citizenship Status and Hypertension, NHIS, 2015–2018. *Note:* The predicted marginals for proportion depressed are from a logistic regression model that included the following covariates: sex, sexual orientation, age, race/ethnicity, BMI, education, self-reported health status, income, years lived in the US, number of chronic conditions, leisure-time physical activity, smoking status, alcohol drinking status, health insurance, employment status, and marital status. Error bars indicate 95% confidence intervals.

Fig. 1, the predicted marginal proportions for hypertension and depression by citizenship status, highlights the interaction effects of citizenship status and hypertension in the logistic regression model. Overall, non-citizens with hypertension had the highest predicted marginal for depression (47.3 %) followed by U.S. citizens with hypertension (39.4 %), citizens without hypertension (38.9 %) and non-citizens without hypertension (37.2 %).

## 4 Discussion

Using nationally representative data from NHIS, this study demonstrates dynamic differences in the presentation of hypertension and depression by citizenship status. To the best of our knowledge, this is the first study to examine the prevalence of hypertension and depression in the general U.S. adult population, and to highlight differences based on citizenship status. Our finding on the prevalence of depression among hypertensive U.S. adults is similar to what has been reported in a systematic review and *meta*-analysis (Li et al., 2015). Of note, both the interaction and predicted marginal analyses consistently indicate that non-U.S. citizens with hypertension had an elevated likelihood of depression compared to U.S. citizens without hypertension although non-citizens had a lower prevalence of depression. Further, the fact that hypertension and citizenship status individually were not significantly associated with depression in the general U.S. adult population implies that both factors (i.e., being a non-citizen and having hypertension) are more likely to play significant roles in predicting depression when they interact but not in isolation. While the underlying factors are not clear, previous studies have reported that non-citizens face many structural barriers such as poverty, lack of health insurance coverage, under treatment, limited access to quality care, and non-adherence to prescription medications compared to U.S.-born citizens (Chen and Vargas-Bustamante, 2011, Guadamuz et al., 2020). A study which utilized the National Health and Nutrition Examination Survey (NHANES) to assess cardiovascular disease risk and treatment by citizenship status indicated that although non-U.S. citizens reported a lower prevalence of hypertension and other chronic conditions, they had considerably lower treatment rates than U.S.-born citizens (Guadamuz et al., 2020). Another study that used the Medical Expenditure Panel Survey and NHIS reported immigrants were less likely to take prescription drugs compared to U.S.-born citizens (Chen and Vargas-Bustamante, 2011). Similarly, the Korea National Health Insurance Claims Database was utilized to assess risk factors of non-adherence to antihypertensive drugs (Cho et al., 2020). The authors reported almost twice the level of non-adherence in immigrants with hypertension compared to native-born Koreans. Moreover, the risk of non-adherence was significantly higher among immigrants without usual source of care and younger adults (Cho et al., 2020).

Health status, sociodemographic and lifestyle factors also play a significant role in depression and other chronic disease outcomes (Akhtar-Danesh and Landeen, 2007, Gabriel et al., 2021, Mann et al., 2022, Villarroel and Terlizzi, 2020). Accordingly, we found that current and former smokers, current and former drinkers, young adults, sexual minorities (i.e., non-heterosexuals), females, non-Hispanic Whites, those who were never married, unemployed, without health insurance, had low income, had more chronic conditions and had poor health were more likely to be depressed. Villarroel et al. analyzed symptoms of depression among U.S. adults, and, similar to our findings, they found young adults aged 18–29 years and women experienced the highest rate of mild, moderate and severe symptoms of depression (Villarroel and Terlizzi, 2020). Living in the U.S. for the longest time (i.e., >15 years) was also associated with higher odds of depression but this was not statistically significant in our study. For certain immigrant groups, length of stay may be an index of higher levels of acculturation which warrants further attention (Divney et al., 2019). Previous studies have suggested that acculturation plays an important role in depression, hypertension and other chronic conditions among migrants (Bernstein et al., 2011, Divney et al., 2019).

On the other hand, our findings indicate a decreased likelihood of depression among racial minorities and adults with low levels of education. Interestingly, insufficient leisure-time physical activity was also associated with lower depression, contradicting existing evidence (Fernandez-Montero et al., 2020). We recommend further studies that focus on several dimensions of leisure-time physical activity including the type, duration and intensity of these activities.

### 4.1 Clinical and research implications

The findings of our study imply that the healthy immigrant paradox, in the context of hypertension-depression prevention and control, may not apply to non-citizens with hypertension. While hypertension marginally increased the odds of depression among the general U.S. population, being a non-U.S. citizen with hypertension increased the risk of depression by 46 %. Thus, clinicians may need to pay more attention to these comorbid conditions, especially when presented among non-citizens. Community-based screenings, currently lacking among immigrant communities, should be encouraged since immigrants and other marginalized populations are less likely to access or use these services despite facing multiple stressors. There is also the need for researchers and clinicians to develop interventions that are culturally appropriate and sensitive to unique cultural practices and identities of specific immigrant sub-populations in the U.S. This can help improve cultural norms for health-seeking behavior and use of medication/treatment among immigrants. Moreover, collaborative efforts are required to address barriers such as limited access to care and non-adherence to medications that are well-known to predispose immigrants to poor health outcomes as well as proper management of these chronic conditions (Cho et al., 2020, Hacker et al., 2015). We further recommend longitudinal studies and evidence-based interventions that take into consideration distinct sociocultural factors of immigrant communities in addition to targeting lifestyle, behavioral risk factors and access to healthcare. Henceforth, some policy changes may be necessary to effectively integrate immigrants into the health care system in light of its rapidly increasing and aging population in the U.S. (Bustamante et al., 2021).

### 4.2 Study limitations and strengths

Our findings should be interpreted with caution due to some limitations. NHIS data is cross-sectional, and therefore we cannot make causal inferences about our findings. Also, the variables we analyzed were self-reported, which may be susceptible to the respondent and social desirability biases and may underestimate our findings. Using complete case analysis to address missing or incomplete data can lead to bias and loss of precision in terms of estimations (Kang, 2013, Mukaka et al., 2016). However, the large sample size utilized in this study was very helpful in approximating accurate estimates and addressing biases in our results. Furthermore, we combined depression frequency and taking medication for depression to determine depression status, which may not be an objective, clinical, or valid measure of depressive symptoms. Nonetheless, this study still presents very important findings about depression among hypertension patients based on U.S. citizenship status since NHIS data is a validated nationally representative survey.

## 5 Conclusion

Non-citizens with hypertension had higher odds of depression compared to U.S. citizens without hypertension, underscoring the critical relationship between citizenship and hypertension in predicting depression. Thus, both citizenship and hypertension are more likely to play significant roles in predicting hypertension when they interact but not in isolation. Community-based screenings and other interventions to address hypertension-depression among non-U.S. citizens should consider the unique cultural practices and healthcare barriers encountered by specific immigrant communities.

## Ethical approval

Not applicable.

## Financial disclosure

No financial disclosures were reported by the authors of this paper.

## Funding

This work is supported by the Division of Intramural Research, National Institute on Minority Health and Health Disparities (ZIA MD000015), Opinions and comments expressed in this article belong to the authors and do not necessarily reflect those of the U.S. Government, Department of Health and Human Services, National Institutes of Health, and National Institute on Minority Health and Health Disparities.

## CRediT authorship contribution statement

**Emmanuel A. Odame:** Conceptualization, Methodology, Visualization, Writing – original draft, Writing – review & editing. **Paul H. Atandoh:** Methodology, Visualization, Writing – original draft, Writing – review & editing. **Lohuwa Mamudu:** Conceptualization, Methodology, Validation, Visualization, Writing – review & editing. **David Adzrago:** Conceptualization, Methodology, Visualization, Writing – original draft, Writing – review & editing. **Ishmael Tagoe:** Conceptualization, Methodology, Visualization, Writing – original draft, Writing – review & editing. **Saanie Sulley:** Conceptualization, Methodology, Visualization, Writing – original draft, Writing – review & editing. **Maureen Boms:** Writing – original draft. **Erasmus Tetteh-Bator:** Writing – review & editing. **Timothy S. McNeel:** Data curation, Formal analysis, Visualization, Writing – review & editing. **Faustine Williams:** Conceptualization, Methodology, Visualization, Writing – original draft, Writing – review & editing, Supervision.

## Declaration of Competing Interest

The authors declare that they have no known competing financial interests or personal relationships that could have appeared to influence the work reported in this paper.

## References

[b0005] Akhtar-Danesh N., Landeen J. (2007). Relation between depression and sociodemographic factors. Int. J. Ment. Health Syst..

[b0010] Bernstein K.S., Park S.Y., Shin J., Cho S., Park Y. (2011). Acculturation, discrimination and depressive symptoms among Korean immigrants in New York City. Commun. Ment. Health J..

[b0015] Blewett A.L., D.J.A.R., King M.L., William K.C.W., Del Ponte N., Convey P., 2021. IPUMS Health Surveys: National Health Interview Survey.

[b0020] Breeden M., Brieler J., Salas J., Scherrer J.F. (2018). Antidepressants and Incident Hypertension in Primary Care Patients. J. Am. Board Fam. Med..

[b0025] Bustamante A.V., Chen J., Felix Beltran L., Ortega A.N. (2021). Health Policy Challenges Posed By Shifting Demographics And Health Trends Among Immigrants To The United States. Health Aff. (millwood).

[b0030] Calvi A., Fischetti I., Verzicco I., Belvederi Murri M., Zanetidou S., Volpi R., Coghi P., Tedeschi S., Amore M., Cabassi A. (2021). Antidepressant drugs effects on blood pressure. Front. Cardiovasc. Med..

[b0035] Centers for Disease Control and Prevention, 2021a. Estimated Hypertension Prevalence, Treatment, and Control Among U.S. Adults.

[b0040] Centers for Disease Control and Prevention, 2021b. Facts About Hypertension.

[b0045] Chang C.D. (2019). Social Determinants of Health and Health Disparities Among Immigrants and their Children. Curr. Probl. Pediatr. Adolesc. Health Care.

[b0050] Chen J.A., Shapero B.G., Trinh N.T., Chang T.E., Parkin S., Alpert J.E., Fava M., Yeung A.S. (2016). Association Between Stigma and Depression Outcomes Among Chinese Immigrants in a Primary Care Setting. J. Clin. Psychiatry.

[b0055] Chen J., Vargas-Bustamante A. (2011). Estimating the effects of immigration status on mental health care utilizations in the United States. J. Immigr. Minor. Health.

[b0060] Cho H., Jeong S., Kang C., Kang H.J., Jang S. (2020). Risk Factors and the Usual Source of Care on Non-Adherence to Antihypertensive Drugs in Immigrants with Hypertension. Patient Prefer. Adherence.

[b0065] Commodore-Mensah Y., Turkson-Ocran R.A., Foti K., Cooper L.A., Himmelfarb C.D. (2021). Associations Between Social Determinants and Hypertension, Stage 2 Hypertension, and Controlled Blood Pressure Among Men and Women in the United States. Am. J. Hypertens..

[b0070] de Mestral C., Stringhini S. (2017). Socioeconomic Status and Cardiovascular Disease: an Update. Curr. Cardiol. Rep..

[b0075] DeJean D., Giacomini M., Vanstone M., Brundisini F. (2013). Patient experiences of depression and anxiety with chronic disease: a systematic review and qualitative meta-synthesis. Ont. Health Technol. Assess. Ser..

[b0080] Divney A.A., Echeverria S.E., Thorpe L.E., Trinh-Shevrin C., Islam N.S. (2019). Hypertension Prevalence Jointly Influenced by Acculturation and Gender in US Immigrant Groups. Am. J. Hypertens..

[b0085] Fernandez-Montero A., Moreno-Galarraga L., Sanchez-Villegas A., Lahortiga-Ramos F., Ruiz-Canela M., Martinez-Gonzalez M.A., Molero P. (2020). Dimensions of leisure-time physical activity and risk of depression in the “Seguimiento Universidad de Navarra” (SUN) prospective cohort. BMC Psychiatry.

[b0090] Gabriel A., Zare H., Jones W., Yang M., Ibe C.A., Cao Y., Balamani M., Gaston M., Porter G. (2021). Evaluating Depressive Symptoms Among Low-Socioeconomic-Status African American Women Aged 40 to 75 Years With Uncontrolled Hypertension: A Secondary Analysis of a Randomized Clinical Trial. JAMA Psychiat..

[b0095] Ginty A.T., Carroll D., Roseboom T.J., Phillips A.C., de Rooij S.R. (2013). Depression and anxiety are associated with a diagnosis of hypertension 5 years later in a cohort of late middle-aged men and women. J. Hum. Hypertens..

[b0100] Grimsrud A., Stein D.J., Seedat S., Williams D., Myer L. (2009). The association between hypertension and depression and anxiety disorders: results from a nationally-representative sample of South African adults. PLoS One.

[b0105] Guadamuz J.S., Durazo-Arvizu R.A., Daviglus M.L., Calip G.S., Nutescu E.A., Qato D.M. (2020). Citizenship Status and the Prevalence, Treatment, and Control of Cardiovascular Disease Risk Factors Among Adults in the United States, 2011–2016. Circ. Cardiovasc. Qual. Outcomes.

[b0110] Guadamuz J.S., Durazo-Arvizu R.A., Daviglus M.L., Calip G.S., Nutescu E.A., Qato D.M. (2021). Statin nonadherence in Latino and noncitizen neighborhoods in New York City, Los Angeles, and Chicago, 2012–2016. J. Am. Pharm. Assoc..

[b0115] Hacker K., Anies M., Folb B.L., Zallman L. (2015). Barriers to health care for undocumented immigrants: a literature review. Risk Manag. Healthc. Policy.

[b0120] Hafner S., Baumert J., Emeny R.T., Lacruz M.E., Bidlingmaier M., Reincke M., Ladwig K.H. (2013). Hypertension and depressed symptomatology: a cluster related to the activation of the renin-angiotensin-aldosterone system (RAAS). Findings from population based KORA F4 study. Psychoneuroendocrinology.

[b0125] Hamad R., Penko J., Kazi D.S., Coxson P., Guzman D., Wei P.C., Mason A., Wang E.A., Goldman L. (2020). Association of Low Socioeconomic Status With Premature Coronary Heart Disease in US Adults. JAMA Cardiol..

[b0130] Ho A.K., Thorpe C.T., Pandhi N., Palta M., Smith M.A., Johnson H.M. (2015). Association of anxiety and depression with hypertension control: a US multidisciplinary group practice observational study. J. Hypertens..

[b0135] Jordan, M., Gebeloff, R., 2022. Amid Slowdown, Immigration Is Driving U.S. Population Growth.

[b0140] Kang H. (2013). The prevention and handling of the missing data. Korean J. Anesthesiol..

[b0145] Kessing L.V., Rytgaard H.C., Ekstrom C.T., Torp-Pedersen C., Berk M., Gerds T.A. (2020). Antihypertensive drugs and risk of depression: a nationwide population-based study. Hypertension.

[b0150] Kochanek K.D., Murphy S.L., Xu J., Arias E. (2019). Deaths: Final Data for 2017. Natl. Vital Stat. Rep..

[b0155] Li Y., Fan Y., Sun Y., Alolga R.N., Xiao P., Ma G. (2021). Antihypertensive Drug Use and the Risk of Depression: A Systematic Review and Network Meta-analysis. Front. Pharmacol..

[b0160] Li Z., Li Y., Chen L., Chen P., Hu Y. (2015). Prevalence of depression in patients with hypertension: a systematic review and meta-analysis. Medicine (Baltimore).

[b0165] Maatouk I., Herzog W., Bohlen F., Quinzler R., Lowe B., Saum K.U., Brenner H., Wild B. (2016). Association of hypertension with depression and generalized anxiety symptoms in a large population-based sample of older adults. J. Hypertens..

[b0170] Mann A., Chan A., Rohatgi A., Caesar M.A., Obedin-Maliver J., Kapp D.S. (2022). Comparison of depressive symptoms and inflammation between sexual minorities and heterosexuals using NHANES study of 8538 participants. Sci. Rep..

[b0175] Marazziti D., Rutigliano G., Baroni S., Landi P., Dell'Osso L. (2014). Metabolic syndrome and major depression. CNS Spectr..

[b0180] Mukaka M., White S.A., Terlouw D.J., Mwapasa V., Kalilani-Phiri L., Faragher E.B. (2016). Is using multiple imputation better than complete case analysis for estimating a prevalence (risk) difference in randomized controlled trials when binary outcome observations are missing?. Trials.

[b0185] Murphy S.L., Xu J., Kochanek K.D., Arias E., Tejada-Vera B. (2021). Deaths: Final Data for 2018. Natl. Vital Stat. Rep..

[b0190] Nambiar L., LeWinter M.M., VanBuren P.C., Dauerman H.L. (2020). Decade-Long Temporal Trends in U.S. Hypertension-Related Cardiovascular Mortality. J. Am. Coll. Cardiol..

[b0195] National Center for Health Statistics, 2019c. National Health Interview Survey, 2018. Public-use data file and documentation.

[b0200] National Center for Health Statistics, 2019a. 2018 National Health Interview Survey (NHIS) Public Use Data Release, in: Statistics, D.o.H.I. (Ed.).

[b0205] National Center for Health Statistics, 2019b. Multiple Imputation of Family Income and Personal Earnings in the National Health Interview Survey: Methods and Examples.

[b0210] Parsons V.L., Moriarity C., Jonas K., Moore T.F., Davis K.E., Tompkins L. (2014). Design and estimation for the national health interview survey, 2006–2015. Vital Health Stat..

[b0215] Ross J., Hua S., Perreira K.M., Hanna D.B., Castañeda S.F., Gallo L.C., Penedo F.J., Tarraf W., Hernandez R. (2019). Association between immigration status and anxiety, depression, and use of anxiolytic and antidepressant medications in the Hispanic Community Health Study/Study of Latinos. Ann. Epidemiol..

[b0220] Rubio-Guerra A.F., Rodriguez-Lopez L., Vargas-Ayala G., Huerta-Ramirez S., Serna D.C., Lozano-Nuevo J.J. (2013). Depression increases the risk for uncontrolled hypertension. Exp. Clin. Cardiol..

[b0225] Stanek M., Requena M., Del Rey A., Garcia-Gomez J. (2020). Beyond the healthy immigrant paradox: decomposing differences in birthweight among immigrants in Spain. Global Health.

[b0230] Villarroel, M.A., Terlizzi, E.P., 2020. Symptoms of Depression Among Adults: United States, 2019. NCHS Data Brief:1-8. 33054920

[b0235] Wang L., Li N., Heizhati M., Li M., Yang Z., Wang Z., Abudereyimu R. (2021). Association of Depression with Uncontrolled Hypertension in Primary Care Setting: A Cross-Sectional Study in Less-Developed Northwest China. Int. J. Hypertens..

[b0240] Wiehe M., Fuchs S.C., Moreira L.B., Moraes R.S., Pereira G.M., Gus M., Fuchs F.D. (2006). Absence of association between depression and hypertension: results of a prospectively designed population-based study. J. Hum. Hypertens..

